# Photocurrent detection of chemically tuned hierarchical ZnO nanostructures grown on seed layers formed by atomic layer deposition

**DOI:** 10.1186/1556-276X-7-290

**Published:** 2012-06-06

**Authors:** Seokhwan Bang, Seungjun Lee, Youngbin Ko, Joohyun Park, Seokyoon Shin, Hyungtak Seo, Hyeongtag Jeon

**Affiliations:** 1 Division of Materials Science and Engineering, Hanyang University, Seongdong-gu, Seoul, 133-791, South Korea; 2 Department of Materials Science & Engineering, Ajou University, Woncheon-Dong, Yeongtong-Gu, Suwon, 443-749, South Korea

**Keywords:** ZnO, nanorod, nanosheet, ALD, hydrothermal growth, band structure, photocurrent, 61.46.Km, 78.67.Qa, 81.07.-b

## Abstract

We demonstrate the morphological control method of ZnO nanostructures by atomic layer deposition (ALD) on an Al_2_O_3_/ZnO seed layer surface and the application of a hierarchical ZnO nanostructure for a photodetector. Two layers of ZnO and Al_2_O_3_ prepared using ALD with different pH values in solution coexisted on the alloy film surface, leading to deactivation of the surface hydroxyl groups. This surface complex decreased the ZnO nucleation on the seed layer surface, and thereby effectively screened the inherent surface polarity of ZnO. As a result, a 2-D zinc hydroxyl compound nanosheet was produced. With increasing ALD cycles of ZnO in the seed layer, the nanostructure morphology changes from 2-D nanosheet to 1-D nanorod due to the recovery of the natural crystallinity and polarity of ZnO. The thin ALD ZnO seed layer conformally covers the complex nanosheet structure to produce a nanorod, then a 3-D, hierarchical ZnO nanostructure was synthesized using a combined hydrothermal and ALD method. During the deposition of the ALD ZnO seed layer, the zinc hydroxyl compound nanosheets underwent a self-annealing process at 150 °C, resulting in structural transformation to pure ZnO 3-D nanosheets without collapse of the intrinsic morphology. The investigation on band electronic properties of ZnO 2-D nanosheet and 3-D hierarchical structure revealed noticeable variations depending on the richness of Zn-OH in each morphology. The improved visible and ultraviolet photocurrent characteristics of a photodetector with the active region using 3-D hierarchical structure against those of 2-D nanosheet structure were achieved.

## Background

Nanostructured materials, which are defined as materials with structural elements, such as clusters, crystallites or molecules, with dimensions in the 1 to 100-nm range, have been the interest of both academic and industrial fields over the past few decades because nanosize scaling allows materials to exhibit novel and significantly improved physical, chemical, and biological properties [1-3]. In addition, nanostructures can provide unprecedented understanding on materials and devices. For these reasons, fabrication methods and characterizations of various nanostructured materials have been extensively investigated. Zinc oxide (ZnO), with a wide band gap (3.37 eV), has been actively studied due to its excellent chemical/electrical/optical properties and the ease of nanostructure growth applicable to nanoscale functional devices, such as sensors, solar cells, light emitting diodes, and ultraviolet lasers [4-7]. It is important to fabricate and control the nanostructure size, density, and shape to produce ZnO nanostructures for specific purposes. ZnO nanostructures in the shapes of rods, belts, nails, tubes, stars, and flowers have been prepared by the thermal evaporation of zinc powder and hydrothermal synthesis [8-12]. Metal organic chemical vapor deposition, spray pyrolysis, laser ablation, sputter deposition, and template-assisted growth synthesis methods are typically employed for these nanostructures. In particular, synthesis of ZnO via a chemical solution route provides an easy and convenient method and is very effective for scale-up, even at low temperature. In this hydrothermal method, various shapes and dimensions of ZnO nanostructures can be obtained by tuning the pH, process temperature, concentration of precursors, and seed layer [13-16]. Among them, the seed layer plays an important role in promoting high-density nucleation through reduction of the thermodynamic barrier [16]. Although previous results have demonstrated methods for successful control of the ZnO nanostructure shape, there are additional challenging factors in ZnO nanostructure growth such as (a) preparation of a pure ZnO chemical composition without undesired element incorporation from a seed layer and (b) production of preferential structural growth over large areas [17-19]. The latter factor is crucial to maximize reactive sites of ZnO in specific orientations for surface chemical applications (e.g., gas sensor and heterogeneous catalysis supports). In this regard, achievement of large areas with a (100) ZnO surface orientation is very useful as it has reactive O- and Zn-polar sites [20-22]; however, this part remains a technical challenge in ZnO nanostructure.

In this study, we reported a change in chemical and physical properties of the ZnO nanostructure as shaping ZnO from two-dimensional (2-D) nanosheet to one-dimensional (1-D) nanorod by tuning the Al_2_O_3_/ZnO thin bilayer film of the seed layer. The preparation of 2-D ZnO nanostructures is still challenging as ZnO exhibits structural polarity, which induces highly anisotropic *c*-axis oriented growth. To create various shapes of ZnO nanostructures, thin Al_2_O_3_ was applied to screen the inherent polarity of ZnO to serve as an amorphizing layer [19]. The seed layers were deposited by atomic layer deposition (ALD) to precisely control the seed layer. ALD is a thin film growth technique that is based on self-limiting and surface reactions, resulting in films deposited in a layer-by-layer fashion. These features can offer the unique capability to coat complex 3-D nanostructure substrates with a precise and conformal layer, even if prepared at low processing temperatures. We finally introduce a combined ALD and hydrothermal synthesis approach by systematic assembly of 3-D hierarchical nanostructures, constructed using sequential loading of nanorods on nanosheets, revealing finely tuned ZnO-like chemical and physical properties by suppressing hydroxyl groups. These 3-D hierarchical nanostructures are proven to enhance the sensitivity of nanoscale ZnO optical sensor greatly.

## Methods

The formation of seed layers was performed on a 100-nm-thick SiO_2_/Si wafer by ALD. First, 5-nm-thick Al_2_O_3_ was deposited using trimethylaluminum (Al(CH_3_)_3_) and deionized water (H_2_O) as Al and oxidant precursors, respectively. Then, 1-, 6-, 12-, and 18-nm-thick ZnO films were deposited on 5-nm-thick ALD Al_2_O_3_ film using diethylzinc (Zn(CH_2_CH_3_)_2_) and deionized water as Zn and oxidant precursors, respectively. Argon was used as a carrier and purge gas. The process temperature and pressure were 150 °C and 0.5 Torr, respectively. The growth rates were 0.1 for Al_2_O_3_ and 0.2 nm/cycle for ZnO films. After coating SiO_2_ substrates with ZnO on Al_2_O_3_ by ALD at various thicknesses, hydrothermal growth of ZnO was performed by suspending the sample upside-down in a Teflon beaker filled with an equimolar aqueous solution (0.02 M) of zinc nitrate hexahydrate (Zn(NO_3_)_2_ · 6H_2_O, 99.0% purity; Sigma Aldrich, Seoul, South Korea) and hexamethylenetetramine (HMT, C_6_H_12_N_4_, 99.0% purity; Sigma Aldrich). Before introducing the substrate into the growth solution, the Teflon beaker containing the precursor solution was maintained in a laboratory oven at 90 °C for 1 hr to reduce the density of free-floating ZnO nanoparticulates. The substrate was then placed in a heated solution and held at the same temperature for 2 hr. At the end of the growth period, the sample was removed from the solution, then immediately rinsed with deionized water to remove residual salt from the surface. Finally, the sample was dried naturally in laboratory air at room temperature. Therefore, the observed changes in the ZnO nanostructure shape were only related to the changes in the seed layer surface produced by control of the ALD ZnO thickness. The morphological characterizations were obtained using a field emission scanning electron microscope (S-4800, Hitachi, Seoul, South Korea) and transmission electron microscopy (TEM, JEM-3010TEM; JEOL Ltd., Akishima, Tokyo, Japan). The crystal structures were determined by X-ray diffraction (XRD, DMAX-2500; Rigaku, Corporation, Tokyo, Japan) with Cu Kα radiation, and the changes in the chemical bonds of the ZnO nanostructures were analyzed using X-ray photoelectron spectroscopy (XPS, ESCA Lab-2220I; VG Semicon, East Grinstead, Weat Sussex, UK) with a Mg source. The binding energy of each element was calibrated using C-C bonds (284.5 eV) in the C 1 s binding state. The optical property is characterized using a UV–VIS spectrophotometer (U-3010, Hitachi). In order to investigate the photo responsibility, 100-nm-thick SiO_2_/Si wafers were used as the substrate for photodetector fabrication. Al_2_O_3_/ZnO (50/10 nm) channel layers were deposited by ALD. Channel layers were patterned by the lift-off method. Metal electrodes comprised of Ti/Au (30/100 nm) were deposited by an e-beam evaporator. The width and length of the channel layer were 400 and 100 μm, respectively. Next, nanostructures were synthesized on the channel layer. The electrical characteristics of individual nanostructure photodetectors were measured by an Agilent B1500A semiconductor analyzer (Agilent Technologies Inc., Santa Clara, CA, USA).

## Results and discussion

**Figure 1:**
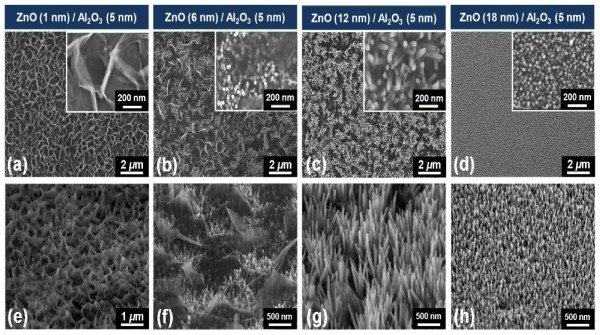
**Typical SEM images of the ZnO nanostructure synthesized on various ALD-grown seed layers.** The thickness of ALD-grown Al_2_O_3_ was fixed at 5 nm, on (**a**) ZnO/Al_2_O_3_ (1 nm/5 nm), (**b**) ZnO/Al_2_O_3_ (6 nm/5 nm), (**c**) ZnO/Al_2_O_3_ (12 nm/5 nm), and (**d**) ZnO/Al_2_O_3_ (18 nm/5 nm). The tilted (30°) views of SEM images are shown in (**e**), (**f**), (**g**), and (**h**), respectively. Inset figures (**a**) to (**d**) were magnified images.

**Figure 2:**
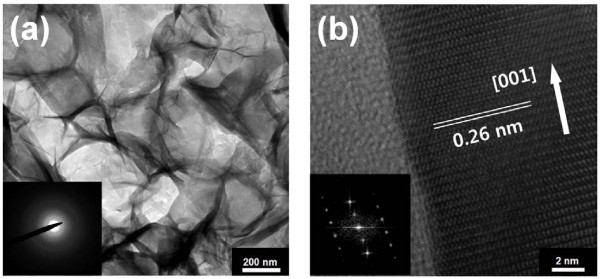
**HRTEM images of nanosheet and nanorod.** (**a**) Nanosheet grown on a ZnO/Al_2_O_3_ (1 nm/5 nm) seed layer and (**b**) nanorod grown on a ZnO/Al_2_O_3_ (18 nm/5 nm) seed layer. The figure insets are diffraction patterns for each HRTEM image.

**Figure 3:**
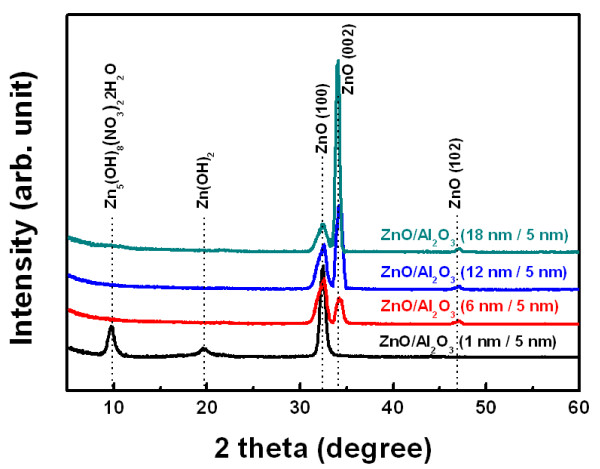
XRD patterns of the ZnO nanostructures synthesized on different ALD-grown seed layers.

Figure 1 shows the SEM images of ZnO nanostructures synthesized on various ALD-grown seed layers. The thickness of the ALD-grown Al_2_O_3_ was fixed at 5 nm, but the thickness of ZnO was varied from 1 to18 nm by changing the number of ALD cycles. In the case of the ZnO/Al_2_O_3_ (1 nm/5 nm) seed layer, a 2-D homogenous film was uniformly formed in a network over the entire region (Figure 1a). The magnified inset image in Figure 1a indicates ultrathin 2-D nanosheets (with a thickness < 10 nm) interlaced with a curved anomalous morphology. As the thickness of the ALD ZnO film in the seed layer increased, the 2-D nanosheet coexisted with a 1-D nanorod. For an ALD ZnO thickness > 18 nm, dense 1-D nanorods grew without the 2-D nanosheet. Figure 2 illustrates the TEM images of the 2-D nanosheet and 1-D nanorods. As shown in Figure 2a, the 2-D nanosheet exhibited a cloud-like sheet, with no specific orientation. The electron diffraction pattern exhibited weak and diffused rings, indicating a polycrystalline structure consisting of small nanocrystalites. In contrast, the 1-D nanorods showed a single crystal phase in Figure 2b. The inset figure in Figure 2b is the corresponding fast Fourier transform of a nanorod, which was indexed to the hexagonal wurtzite structure of ZnO along the [0001] zone axis. The crystal structures of the ZnO nanostructure were characterized to further investigate the effect of the seed layer. Figure 3 represents the XRD pattern of the ZnO nanostructure synthesized on different seed layers. The diffraction pattern of the ZnO nanostructure on a ZnO/Al_2_O_3_ (1 nm/5 nm) seed layer exhibited peaks at 2θ = 9.6°, representing zinc hydroxide nitrate dihydrate (Zn_5_(OH)_8_(NO_3_)_2_·2H_2_O; JCPDS:25–1028); 2θ = 19.8°, representing zinc hydroxide (Zn(OH)_2_; JCPDS:38–0385); and 2θ = 31.8°, representing ZnO (JCPDS:36–1451). Therefore, the XRD pattern indicates that the ZnO nanostructure on the ZnO/Al_2_O_3_ (1 nm/5 nm) seed layer was not pure but was a mixture of zinc oxide, zinc hydroxide nitrate dehydrate, and zinc hydroxide. As the ZnO thickness in the seed layer increases, peaks related to the zinc hydroxyl compound disappear, and only ZnO-related peaks at 2θ = 31.9° and 34.3° corresponding to (100) and (002), respectively, were observed. Specifically, the relative intensity of the (002) peak, the preferred growth direction of pure ZnO, becomes more intense.

**Figure 4:**
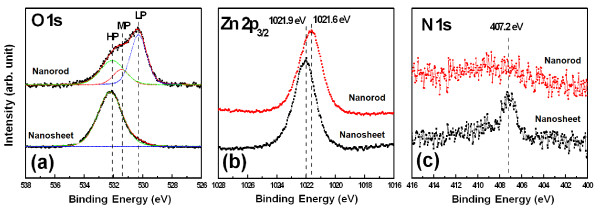
**XPS spectra.** (**a**) O 1 s, (**b**) Zn 2p_3/2_, and (**c**) N 1 s for the synthesized ZnO nanostructures corresponding to the nanosheet grown on a ZnO/Al_2_O_3_ (1 nm/5 nm) seed layer and nanorod grown on a ZnO/Al_2_O_3_ (18 nm/5 nm) seed layer. The figure insets are diffraction patterns for each HRTEM image. The deconvoluted peaks corresponding to LP, MP, and HP components are shown as green, red, and blue peaks in (**a**), respectively.

The surface chemical characteristics of the ZnO nanostructures were analyzed by XPS. The XPS spectra of the ZnO nanosheet and nanorod are shown in Figure 4. The O 1 s peaks of Figure 4a can be deconvoluted into three peaks corresponding to the low binding energy (LP), middle binding energy (MP), and high binding energy (HP) components centered at 530.30 ± 0.1, 531.41 ± 0.11, and 532.45 ± 0.05 eV, respectively. The LP at 530.30 ± 0.1 eV was attributed to O^2−^ ions surrounded by Zn in the ZnO compound system, serving as an indicator of the amount of oxygen atoms in a fully oxidized, stoichiometric environment [23]. The MP, centered at 531.50 ± 0.1 eV, is associated with O^x−^ ions (*x* < 2) in the oxygen-deficient regions within the ZnO matrix and is related to oxygen vacancies [24]. The HP, located at 532.50 ± 0.1 eV, is typically attributed to chemisorbed oxygen, dissociated oxygen, or OH^−^ groups on the surface. In this system, the HP corresponds to zinc hydroxyl compounds, such as Zn_5_(OH)_8_(NO_3_)_2_·2H_2_O and Zn(OH)_2_[25,26]. The O 1 s core binding energy of the nanosheet was 532.3 eV, and that of the nanorod was 530.7 eV. This difference in the O 1 s peak position implies that the surface chemical composition of the nanosheet was different from that of the nanorod. The XPS spectra of the O 1 s peak were deconvoluted using a combination of Gaussian (80%) and Lorentzian (20%) fitting, and an area ratio was calculated for the three LP, MP, and HP peaks. The nanorod area ratio of HP / (LP + MP + HP) was 28.7. It was postulated that hydrothermal synthesis of ZnO in an aqueous solution and the subsequent washing in water naturally produces Zn(OH)_2_ through the reaction with hydroxyl group, and OH/H_2_O molecules in air were also chemisorbed to the ZnO nanorod surface during air drying. Alternatively, the nanosheet area ratio of HP / (LP + MP + HP) was 97.1, suggesting that the nanosheet consisted of zinc hydroxyl bonds, which should be distinguished from the chemisorbed H_2_O molecules in air. The difference in the initial nucleation and growth process causes this difference in the chemical composition. Figure 4b shows the Zn 2p_3/2_ XPS spectra for the nanorod and nanosheet. The Zn 2p_3/2_ binding energy of the nanosheet (1,021.9 eV) is greater than that of the nanorod (1,021. 6 eV), which indicates that the nanosheet is more oxidized than the nanorod. In addition to the prominent O 1 s and Zn 2p_3/2_ spectra, we also observed the N 1 s spectra of ZnO nanostructures and a weak peak, centered near 407.2 eV, corresponding to nitrate, in the nanosheet [27] but not in the nanorod. None of the binding states in the Al 2p spectra was detected in either the nanosheet or nanorod. The XPS results support that the composition of the nanosheet consisted of ZnO, Zn(OH)_2_ and Zn_5_(OH)_8_(NO_3_)_2_·2H_2_O, corroborating the XRD data in Figure 3. Generally, the growth mechanism of ZnO nanorods in the zinc nitrate-hexamethylenetetramine system is as follows:

(1) $${\left({\text{CH}}_{2}\right)}_{6}{\text{N}}_{4}+{6\text{H}}_{2}\text{O}\to 6\text{HCHO}+{4\text{NH}}_{3}$$

(2) $${\text{NH}}_{3}+{\text{H}}_{2}\text{O}↔{{\text{NH}}_{4}}^{+}+{\text{OH}}^{-}$$

(3) $$\text{Zn}{\left({\text{NO}}_{3}\right)}_{2}\cdot {6\text{H}}_{2}\text{O}\to {\text{Zn}}^{2+}+2{\left({\text{NO}}_{3}\right)}^{-}+{\text{6 H}}_{2}\text{O}$$

(4) $${\text{Zn}}^{2+}+{2\text{OH}}^{-}↔\text{Zn}{\left(\text{OH}\right)}_{2}↓↔\text{ZnO}↓+{\text{H}}_{2}\text{O}$$

(5) $${5\text{Zn}}^{2+}+{8\text{OH}}^{-}+{{2\text{NO}}_{3}}^{2-}+{2\text{H}}_{2}\text{O}\to {\text{Zn}}_{5}{\left(\text{OH}\right)}_{8}{\left({\text{NO}}_{3}\right)}_{2}\cdot {2\text{H}}_{2}\text{O}↓$$

(6) $${\text{Zn}}_{5}{\left(\text{OH}\right)}_{8}{\left({\text{NO}}_{3}\right)}_{2}\cdot {2\text{H}}_{2}\text{O}↓\to 5\text{ZnO}+{2\text{HNO}}_{3}+{5\text{H}}_{2}\text{O}$$

Hexamethylenetetramine produced formaldehyde, ammonia, and the hydroxide ions as shown in Equations 1 and 2. As shown in Equation 3, zinc nitrate produces Zn^2+^. As the concentrations of the Zn^2+^ and OH^−^ ions exceed a critical value, the precipitation of ZnO nuclei starts. As shown in Equation 4, Zn(OH)_2_ can be transformed into ZnO crystals via chemical reactions. The precipitates of Zn(OH)_2_ are more soluble than the ZnO precipitates; therefore, the Zn(OH)_2_ precipitates continuously produce Zn^2+^ and OH^−^ ions, which form the ZnO nuclei. Due to the crystal structure of ZnO, the nuclei have a hexagonal shape, and hexagonal ZnO nanorods grow from the nuclei [16,28]. When the ZnO seed layer is immersed in water, the oxide surface is hydrolyzed and a layer of hydroxide forms. Many metal oxides will hydrolyze in the presence of water to form a hydroxide layer at the surface since water molecules can be both physically and chemically adsorbed on a metal oxide surface. Therefore, the ZnO seed layer surface is charged by the surface amphoteric reaction with H^+^ or OH^−^ ions. This charged surface is favored to attract opposite charges (Zn^+^ or OH^−^) in solution to cover the surface, which would form ZnO. Thus, the dense 1-D ZnO nanorods grow layer by layer, leading to good alignment, as shown in Figure 1d. The ZnO nanostructure on the ZnO/Al_2_O_3_ (1 nm/5 nm) seed layer was a 2-D nanosheet, not 1-D nanorod. This morphology may originate from the formation of Zn_5_(OH)_8_(NO_3_)_2_·2H_2_O. Indeed, metal ions such as Zn^2+^, Co^2+^ and Ni^2+^ form the initial kinetic-driven phases of layered metal hydroxide nitrates in an alkaline medium under hydrothermal conditions, which are easy to crystallize into thin, crumpled sheets because of the layered crystallographic structure [29-31]. A nanosheet phase of Zn_5_(OH)_8_(NO_3_)_2_·2H_2_O was formed by the reaction of Zn^2+^, OH^−^, NO_3_^−^ and H_2_O in an aqueous solution, as shown in Equation 5. The phase of Zn_5_(OH)_8_(NO_3_)_2_·2H_2_O consists of tetrahedral ZnO_4_ and octahedral ZnO_6_ with NO_3_^−^ anions intercalated with the positively charged layers of the hydroxide [Zn_5_(OH)_8_(H_2_O)_2_^2+^ to maintain charge neutrality. This formation of Zn_5_(OH)_8_(NO_3_)_2_·2H_2_O could be caused by a difference in the pH at the seed layer surface and polarity shielding of the Al_2_O_3_ layer. The ZnO surface has an isoelectric point of pH 9.5 in water [32]. The Al_2_O_3_ surface is nearly neutral and has an isoelectric point of pH 7.0 to 7.5 in water [33]. During the transition between ZnO and Al_2_O_3_, ALD ZnO and Al_2_O_3_ hydroxyls will coexist on the alloy film surface. This coexistence may allow a proton exchange surface reaction to occur [34]:

(7) $${\text{AlOH}}^{*}+{\text{ZnOH}}^{*}\to {\text{ZnOH}}^{2+\ldots }{\text{AlO}}^{-*}$$

**Figure 5:**
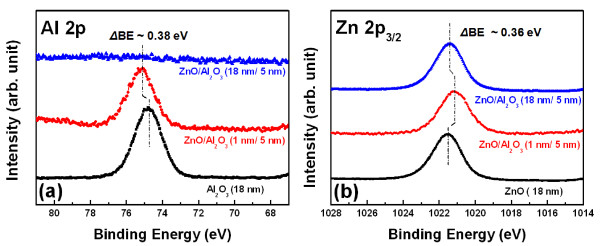
**XPS spectra.** (**a**) Al 2p and (**b**) Zn 2p_3/2_ for ZnO/Al_2_O_3_ (1 nm/5 nm) and ZnO/Al_2_O_3_ (18 nm/5 nm) seed layers, respectively.

The chemical binding states of the ZnO/Al_2_O_3_ seed layers surfaces were investigated by XPS, as shown in Figure 5. The XPS measurements indicate a significant change in the chemical binding at ZnO/Al_2_O_3_ with increasing thickness of the top ZnO layer. The Al 2p and Zn 2p_3/2_ core level spectra of the ZnO/Al_2_O_3_ (18 nm/5 nm) seed layer have binding energies of 1,021.49 and 74.72 eV, respectively. However, the Al 2p and Zn 2p_3/2_ spectra of the ZnO/Al_2_O_3_ (1 nm/ 5 nm) seed layer shifted 0.38 eV toward the high binding energy direction and 0.36 eV toward the low binding energy direction, respectively. Given the electronegativities of Al (1.6) and Zn (1.7), the observed core level shifts are likely due to the charge transfer under a chemical binding process between Al_2_O_3_ and ZnO, where Al tends to donate an electron and Zn tends to gain an electron. Therefore, the XPS results in Figure 5 show a high correlation to the formation of the compound of ZnO and Al_2_O_3_. The formation of the Al_2_O_3_/ZnO complex may deactivate the surface hydroxyl groups (the reaction in Equation 7 is not applicable in this case) and decrease the surface pH, leading to the formation of zinc hydroxide nitrate dehydrate. This surface complex can also render them less reactive to the Zn^+^ or OH^−^ precursors in solution, resulting in a decrease in ZnO nucleation at the seed layer surface. Additionally, a thin amorphous Al_2_O_3_ layer can disrupt the crystalline continuity of the subsequent ALD ZnO and effectively screen the inherent surface polarity of the ZnO [20]. With increasing ALD ZnO cycles, the morphology of the nanostructure changes from 2-D nanosheet to 1-D nanorod because the seed layer surface recovers the natural crystallinity and polarity of ZnO. As shown in Figure 5, the ZnO/Al_2_O_3_ (18 nm/ 5 nm) seed layer exhibits Zn 2p_3/2_ core level spectra at 1,021.50 eV, similar to that of pure 18-nm-thick ALD ZnO (1,021.49 eV), but did not exhibit an Al 2p signal. Thus, the nanorod morphology greatly depends on the substrate surface or seed layer surface, which is considered a key controlling factor.

**Figure 6:**
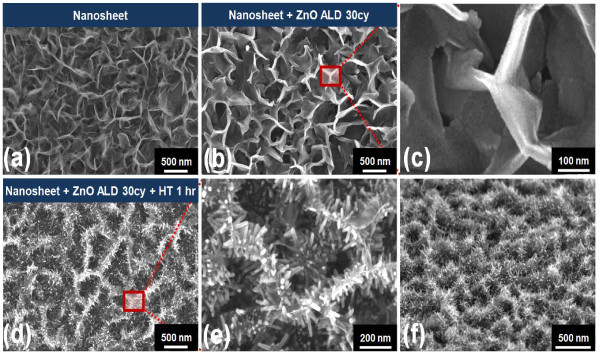
**SEM images of the 3-D hierarchical ZnO synthesized with the combined hydrothermal and ALD method.** (**a**) 2-D nanosheet on a ZnO/Al_2_O_3_ (1 nm/5 nm) seed layer by hydrothermal synthesis, (**b**) 2-D nanosheet after 30 ALD cycles of ZnO deposited at 150 °C, (**c**) magnified SEM image of the red square region in Figure 6b, (**d**) 3-D hierarchical ZnO nanostructure after application of the hydrothermal method for 1 hr, (**e**) magnified SEM image of the red square region in Figure 6d, and (**f**) tilted (30°) view of the 3-D hierarchical ZnO nanostructure.

**Figure 7:**
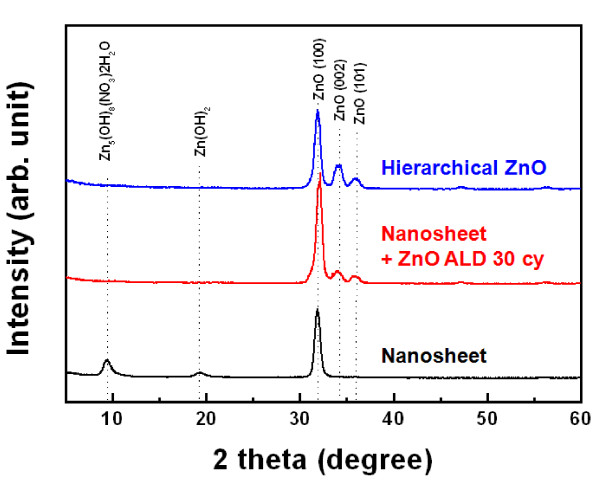
XRD patterns of the nanosheet and ZnO nanostructure synthesized using the combined hydrothermal and ALD method.

A 3-D hierarchical nanostructure was synthesized using a 2-D nanosheet template, as shown in Figure 6. The 2-D nanosheets were first prepared using a ZnO/Al_2_O_3_ (1 nm/5 nm) seed layer as shown in Figure 6a. A ZnO seed layer was then deposited on the 2-D nanosheet with 30 ALD cycles at 150 °C as in Figure 6b. This ALD sequence provides a stable and uniform nucleation layer on the complex, tangled 2-D nanosheet for a second ZnO hydrothermal growth. The magnified SEM image shows that the ALD ZnO seed layer was uniformly deposited on the 2-D nanosheet, as shown in Figure 6c. The hydrothermal process then resulted in uniform growth of the nanorods, normal to the nanosheet surfaces, over the entire exposed surface area. Images of the resultant hierarchical nanosheet/nanorod structures are shown in Figure 6e. The magnified SEM image also indicates that 1-D ZnO nanorods were well aligned on the 2-D nanosheet, as shown in Figure 6f. Figure 7 shows XRD patterns of the ZnO nanostructure synthesized by the combined hydrothermal and ALD method. Note that the sample with the ZnO seed layer deposited on the 2-D nanosheet with 30 ALD cycles at 150 °C did not exhibit Zn_5_(OH)_8_(NO_3_)_2_·2H_2_O or Zn(OH)_2_ XRD peaks at 2θ = 9.6° or 19.8°, respectively. The ZnO-related XRD peaks at 2θ = 34.3° and 36.2° correspond to the (002) and (101) planes, respectively. After the subsequent hydrothermal deposition process (0.02 M; zinc nitrate, HMT), ZnO-related XRD peaks at 2θ = 34.3° and 36.2° corresponding to the (002) and (101) planes intensified. These XRD changes occurred because the thin-layered Zn_5_(OH)_8_(NO_3_)_2_·2H_2_O and Zn(OH)_2_ were unstable in the reaction solution, while ZnO is the final thermodynamically stable phase. Thus, these zinc hydroxyl compounds easily transformed to ZnO during the annealing process at 120 °C to 170 °C based on Equation 9 [35]:

(8) $${\text{Zn}}_{5}{\left(\text{OH}\right)}_{8}{\left({\text{NO}}_{3}\right)}_{2}\cdot {2\text{H}}_{2}\text{O}\to {\text{Zn}}_{3}{\left(\text{OH}\right)}_{4}{\left({\text{NO}}_{3}\right)}_{2}+2\text{ZnO}+{4\text{H}}_{2}\text{O}\to 5\text{ZnO}+{2\text{HNO}}_{3}+{5\text{H}}_{2}\text{O}$$

**Figure 8:**
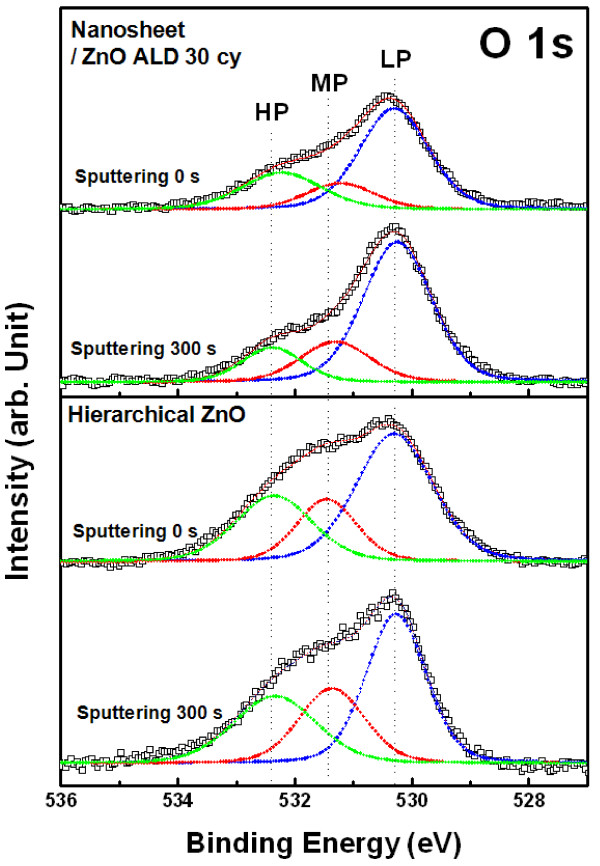
**O 1 s XPS spectra with and without 300-s sputtering and hierarchical ZnO nanostructure.** O 1 s XPS spectra with and without 300-s sputtering of the nanosheet after 30 ALD cycles of ZnO deposited at 150 °C and the hierarchical ZnO nanostructure. The deconvoluted peaks corresponding to LP, MP, and HP components are shown as green, red, and blue peaks, respectively, in all XPS spectra.

During the ZnO seed layer deposition, the zinc hydroxyl compound nanosheet undergoes a self-annealing process at 150 °C, resulting in the formation of a ZnO nanosheet without collapse of its intrinsic morphology. As shown in the ZnO nanostructure XPS spectra in Figure 8, the O 1 s peaks of both the nanosheet after 30 ALD cycles of ZnO at 150 °C and hierarchical ZnO were different from that of the nanosheet (Figure 4a) based on major LP binding states near 530.7 eV. The area ratio of LP / (LP + MP + HP) related to the Zn-O bond was 59.7 for the nanosheet after 30 ALD cycles of ZnO, and 40.3 for the hierarchical ZnO. After argon ion sputtering for 300 s, the ratios were increased to 66.4 for the nanosheet with 30 ALD cycles of ZnO, and 52.9 for hierarchical ZnO. The area ratios of HP / (LP + MP + HP) related to Zn(OH)_2_ concomitantly decreased. Additionally, none of the binding states in the N 1 s spectra were detected in either the nanosheet with ZnO ALD or 3-D hierarchical ZnO, even after argon ion sputtering for 300 s. Therefore, nanosheet compositions, such as the unstable intermediates Zn(OH)_2_ and Zn_5_(OH)_8_(NO_3_)_2_·2H_2_O, can be easily converted to pure ZnO through an ALD process at 150 °C and self-annealing.

**Figure 9:**
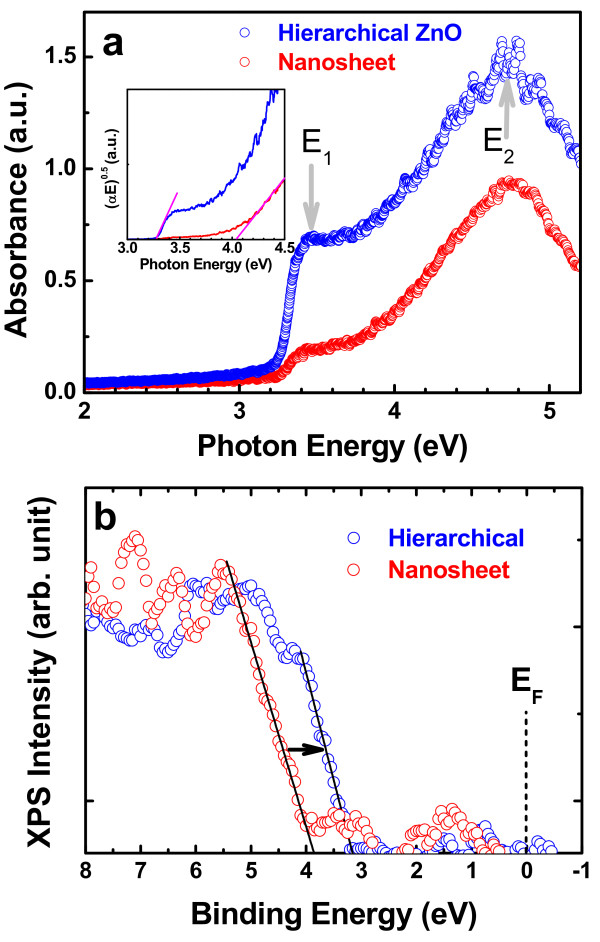
**UV/visible optical absorbance and VB edge XPS spectra.** (**a**) UV/visible optical absorbance spectra and (**b**) VB edge XPS spectra for the nanosheet and hierarchical ZnO nanostructure.

The physical properties such as bandgap and electrical conductivity characteristics of mixed nanostructure zinc hydroxyl compounds are quite different from those of intrinsic ZnO. For example, Zn(OH)_2_ and Zn_5_(OH)_8_(NO_3_)_2_·2H_2_O are typically known as insulators, not semiconductors. We observed that Zn-OH bonds are significantly incorporated to the nanosheet morphology but reduced to the level in typical ZnO for hierarchical ZnO. This morphological chemical variation turned out to reveal the significant physical difference as well. Figure 9a,b shows the UV/visible absorbance spectra and valence band (VB) edge XPS spectra of the nanosheet and hierarchical ZnO, respectively. In Figure 9a, the stronger absorbance intensity in hierarchical ZnO than nanosheet is due to (a) the additional absorption by ZnO nanorod in hierarchical structure and (b) increased optical propagation length due to the multi-reflection between ZnO nanorods. The crucial physical difference between two samples is found from the optical bandgap value. In spite that both samples show the absorption rise at 3.3 eV of photon energy which is a typical direct bandgap of ZnO, the relative absorption intensity at 3.3 eV is much weaker in the nanosheet than in the hierarchical ZnO. The direct bandgap can be found by the extrapolation to the absorption onset edge in the plot of (*α*·*E*_photon_)^2^, where *α* is the absorption coefficient and *E*_photon_ is the photon energy. In the inset of Figure 9a, the absorption onset energy is evidently different depending on samples: 3.3 eV for hierarchical structure and 4 eV for nanosheet. Therefore, it would be more physically meaningful to declare that the nanosheet has a much higher optical bandgap at 4 eV than that in the typical ZnO at 3.3 eV. This is considered due to the modified band electronic structure in the nanosheet by empty Zn 3-D molecular orbital (MO) state mixed with OH. The theoretical calculations indicated that the presence of H/OH will form the higher energy states against the conduction band edge in ZnO, and also, the donation of electron from H to surface O occurs as to increase surface electron density and conductivity [36]. Although it is not certain that the carrier density is increased in the nanosheet since Zn-OH in the nanosheet is not equivalent to the surface OH doping effect in ZnO as in predicted in the calculation, the high direct bandgap of the nanosheet is certainly related to the high-lying Zn-OH MO states. Because the optical absorption in the direct transition is proportional to the joint density of state, the spectral absorption intensity can be used to compare density of states. Two major energy states, E_1_ (3.5 eV) and E_2_ (4.6 eV) were resolved by the second derivation of absorbance spectra. The physical origin of two energy states in ZnO has been assigned as the crystal-field splitting of empty Zn3d-O2p MO states [37]. However, the additional Zn-OH MO states in the nanosheet modify these empty states, and thus, the relative ratio of E_2_ and E_1_ peak intensity is quite different between two samples: 2.1 for the hierarchical structure and 4.5 for the nanosheet. It is interesting to point out that the factors for (a) E_2_/E_1_ peak ratio and (b) HP/HP + MP + LP O 1 s peak ratio (Figure 8) of the nanosheet against those of hierarchical structure are very similar as 2.1 and 1.9, respectively. Therefore, this similarity supports that Zn-OH is responsible for the modified optical absorption in the nanosheet from the typical ZnO nanorod. In similar to the optical absorbance result, VB edge XPS spectra also show the considerable spectral differences. The VB edge onset energy is approximately 3 eV in hierarchical and 3.8 eV of binding energy in nanosheet, respectively. Furthermore, the spectral features above VB edge are noticeably different. In the nanosheet, it is considered that the occupied Zn-OH MO state is responsible for the VB edge shift toward high-binding energy and different VB edge spectra features. Consequently, optical absorbance and XPS VB edge results coherently prove that the band electronic structure in nanosheet containing significant amount of OH groups is markedly different from that in the regular ZnO as expected from the chemical analysis. On the other hand, hierarchical structure shows the recovery of the band electronic structure to that of the regular ZnO while conserving the complex nanorod/nanosheet 3-D structure. Therefore, this unambiguously confirms the conversion of nanosheet from OH-rich ZnO to the regular ZnO in both physical and chemical properties.

**Figure 10:**
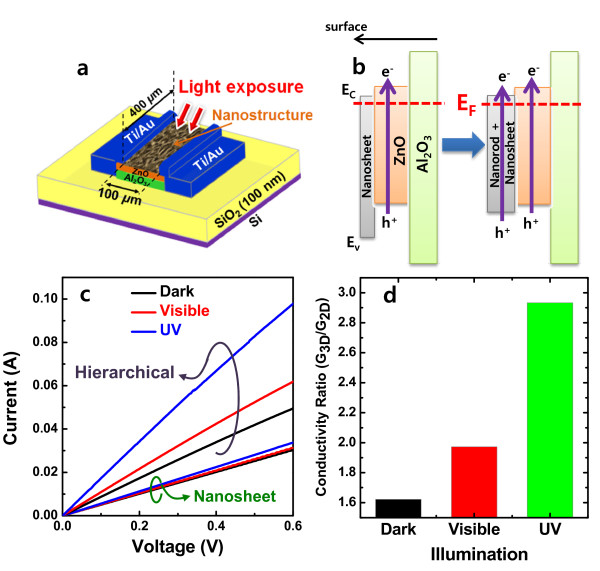
**Schematic illustration of ZnO nanostructure photodetector, electronic band alignment, I-V curves for photodetectors, and conductivity ratio.** (**a**) Schematic illustration of ZnO nanostructure photodetector, (**b**) electronic band alignment of active region consisting of (left) the ZnO nanosheet and (right) the hierarchical ZnO nanostructure on ZnO/Al_2_O_3_ seed layer, (**c**) I-V curves for photodetectors with 2-D nanosheet and 3-D hierarchical nanostructure, and (**d**) conductivity ratio (*G*_3-D_/*G*_2-D_) of 3-D hierarchical nanostructure over 2-D nanosheet under the dark-, visible-, and UV-light illumination conditions.

The highly interesting application of ZnO nanostructure is an optoelectronic device [38,39]. In this study, the OH-free complex 3-D ZnO nanostructure was achieved, and this offers an advanced technical approach for the enhanced optoelectronic application of ZnO nanostructure without the chemical property deterioration as a result of the nanostructure formation. The photodetectors were fabricated to investigate the photoconduction efficiency for nanosheet only (i.e., 2-D structure) and hierarchical structure (i.e., 3-D structure) active regions as shown Figure 10a. The ZnO and Al_2_O_3_ in the seed layer act as the carrier conduction channel and the insulator, respectively. The nanosheet and hierarchical ZnO mainly impact on the light absorption improvement through the internal light scattering/trapping at nanostructures while contributing the partial carrier conduction by the random connection between nanosheets and nanorods at a low fraction of current. Based on physical analysis in Figure 9, the schematic band alignment was constructed as shown in Figure 9b. The nanosheet containing a significant amount of OH groups has a limited optical absorption for visible and UV light because of a high-optical bandgap at approximately 4 eV, but after conversion to ZnO-like nanosheet upon the ZnO nanorod formation in 3-D structure, the optical bandgaps in both nanosheet and nanorod converged to approximately 3.3 eV and visible absorption is also improved. In a support of the enlarged light propagation length and scattering events in nanorods, the photo-induced exciton generation is greatly improved. In addition, the surface electric field through electron capture at the surface O adsorbates in ZnO nanorod leads to the effective charge separation of photo-excited electrons and holes, and thereby reduces the recombination probability of exciton [39]. This combined effect between improved optical absorption and charge separation results in enhancement of photocurrent in the photodetector with 3-D hierarchical ZnO active region as shown in Figure 10c. In contrast to the nanosheet case showing the slight increase only in UV photocurrent, the hierarchical ZnO photodetector revealed photocurrent increase both in visible- and UV-light illumination. In Figure 10d, the conductivity ratio (*G*_3-D_/*G*_2-D_) taken from the photocurrent result of hierarchical (3-D) and nanosheet (2-D) photodetectors was plotted for dark-, visible-, and UV-light illumination conditions. As aforementioned, the dark *G*_3-D_/*G*_2-D_ ratio is slightly increased due to the additional channel area of nanorods by a factor of 1.6, but *G*_3-D_/*G*_2-D_ ratio under light illumination is much higher than that under the dark condition: approximately 100% increase for visible light, and approximately 190% increase against 2-D photodetector. The total conductivity (*G*_total_) in the photodetector is expressed as a sum of dark conductivity (*G*_dark_) and photo conductivity (*G*_photo_):

(9) $$G_{\text{total}}=G_{\text{dark}}+G_{\text{photo}}$$

This, the net *G*_photo_ enhancement in 3-D ZnO photodetector, is 40% increase for the visible light and 130% increase for the UV-light illumination against 2-D photodetector. Thus, the photoconduction efficiency result indicates that the qualitative difference in nanostructures having Zn(OH)_2_ from those with ZnO acts as an important key factor in the ZnO-based optical sensor.

The fabrication of (a) thin and relatively pure 2-D nanosheets without collapse of its intrinsic morphology and (b) 3-D hierarchical ZnO nanostructures compared to nanosheets has the following potential advantages in various fields. The ultrathin ZnO nanosheets can lead to enhanced gas and/or organic molecule adsorption due to the larger specific surface area. Additionally, the thin, sheet-like structures can enhance the transportability of light-induced charges from the surface to the inside due to the limited thickness (<20 nm). With regard to the mechanical properties, the aggregation of porous, net-like arrangements of ZnO nanosheets can be effectively prevented by coating with the dense ALD ZnO films to maintain the original, large active surface area. As an interesting potential application of this finding, the large surface area and interspaces of the 3-D hierarchical ZnO nanostructures may offer improved diffusion and mass transportation of molecules and charges in photochemical reactions.

## Conclusions

The nanostructure morphology change from a 2-D nanosheet to 1-D nanorod was controlled by changing the seed layer surface. During the ALD of the seed layer, ZnO and Al_2_O_3_ hydroxyls coexisted on the alloy film surface, leading to deactivation of the surface hydroxyl groups. This surface complex decreases pure ZnO nucleation on the seed layer surface. Additionally, a thin amorphous Al_2_O_3_ layer disrupts the crystalline continuity of the subsequent ALD ZnO, thereby effectively screening the inherent surface polarity of the ZnO and finally inducing a 2-D zinc hydroxyl compound nanosheet formation on the seed layer. With increasing ALD cycles of ZnO in the seed layer, the morphology of the nanostructure changes from a 2-D nanosheet to 1-D nanorods by recovering the natural crystallinity and polarity of ZnO. Thus, the nanorod morphology greatly depends on the property of the substrate surface or seed layer surface, which is considered a key factor. The thin ALD ZnO nucleation layer conformally covered the knotty nanosheet to produce the nanorod. During the ZnO seed layer formation, a zinc hydroxyl compound nanosheet undergoes self-annealing at 150 °C, resulting in morphological transformation to a pure ZnO nanosheet without the collapse of its intrinsic morphology. This 3-D hierarchical nanostructure revealed finely tuned ZnO-like chemical and physical properties by eliminating hydroxyl groups preexisting 2-D nanosheet. The 3-D hierarchical nanostructures are also proven to improve the sensitivity of nanoscale ZnO-based optical sensor. Therefore, this study demonstrates that ALD is a unique approach for changing the surface polarity of a seed layer and conformal hydrothermal nucleation layer formation, creating complex mixtures of 2-D nanosheets with 1-D nanorods.

## Competing interests

The authors declare that they have no competing interests.

## Authors’ contributions

SB, HS, and HJ designed experiments. SB, SL, YK, JP, SS, and HS carried out the experiments, tested the nanostructures, and fabricated optical sensor devices. SB, HS, and HJ wrote the manuscript. All authors read and approved the final manuscript.
